# Acupuncture Improved Neurological Recovery after Traumatic Brain Injury by Activating BDNF/TrkB Pathway

**DOI:** 10.1155/2017/8460145

**Published:** 2017-01-24

**Authors:** Xiaohong Li, Chong Chen, Xiping Yang, Jingjing Wang, Ming-liang Zhao, Hongtao Sun, Sai Zhang, Yue Tu

**Affiliations:** ^1^Institute of Traumatic Brain Injury and Neurology, Pingjin Hospital, Logistics University of Chinese People's Armed Police Forces, Tianjin 300162, China; ^2^Key Laboratory of Neurotrauma Repair of Tianjin, Tianjin 300162, China

## Abstract

How to promote neural repair following traumatic brain injury (TBI) has long been an intractable problem. Although acupuncture has been demonstrated to facilitate the neurological recovery, the underlying mechanism is elusive. Brain-derived neurotrophic factor (BDNF) exerts substantial protective effects for neurological disorders. In this study, we found that the level of BDNF and tropomyosin receptor kinase B (TrkB) was elevated spontaneously after TBI and reached up to the peak at 12 h. Nevertheless, this enhancement is quickly declined to the normal at 48 h. After combined stimulation at the acupoints of Baihui, Renzhong, Hegu, and Zusanli, we found that BDNF and TrkB were still significantly elevated at 168 h. We also observed that the downstream molecular p-Akt and p-Erk1/2 were significantly increased, suggesting that acupuncture could persistently activate the BDNF/TrkB pathway. To further verify that acupuncture improved recovery through activating BDNF/TrkB pathway, K252a (specific inhibitor of TrkB) was treated by injection stereotaxically into lateral ventricle. We observed that K252a could significantly prevent the acupuncture-induced amelioration of motor, sensation, cognition, and synaptic plasticity. These data indicated that acupuncture promoted the recovery of neurological impairment after TBI by activating BDNF/TrkB signaling pathway, providing new molecular mechanism for understanding traditional therapy of acupuncture.

## 1. Introduction

Traumatic brain injury (TBI) is a leading cause of death and disability worldwide, particularly among children and young adults. How to promote neural repair following TBI has long been an urgent and intractable problem for the medical community [1, 2]. From the traditional Chinese medicine point of view, the overall principle of treatment for TBI is to promote blood circulation to remove blood stasis, phlegming resuscitation, and dredging the channel. As one of the most important treatments, acupuncture could stimulate the surface acupoint and regulate the function of Qi, blood, and organs, which could strengthen the body resistance to eliminate pathogenic factors and equilibrium between yin and yang [3].

As a treatment of traditional Chinese medicine, acupuncture mechanically stimulates specific acupoints with fine needles. Although the efficacy has been demonstrated, the biological basis of acupuncture remains to be elucidated [4, 5]. Previous studies found that acupuncture could significantly improve the ischemia and hypoxia after TBI and promote the regeneration of nerve in injured tissues [6–8]. These studies suggested that acupuncture could facilitate the recovery of TBI and give assistance to the growth factors [9, 10]. However, the underlying mechanism of acupuncture repairing neurological function is elusive.

Neurotrophic factors had been verified to activate repair mechanisms and stimulate neuroregeneration [11, 12]. As one of the most important neurotrophic factors, brain-derived neurotrophic factor (BDNF) is a key regulator of synaptic connections, synaptic plasticity, and neural survival and growth, playing an important role in rebuilding construction and function [13–15]. However, the question of short half life period, insufficient endogenous number, and difficulty to penetrate blood brain barrier limit the clinical application of BDNF. Therefore, finding effective measures that could not only exert and amplify cerebral protective effect of BDNF but also overcome the above difficulties is essential. The role and underlying mechanism of acupuncture on BDNF repairing TBI is unclear. We hypothesized that modulation of BDNF may be the underlying mechanism by which stimulation of acupuncture exerts its neuroprotective effect. Therefore, in the present study, the therapeutic efficacy of acupuncture against neurological deficits was evaluated in TBI rats, and the effects of acupuncture on BDNF expression and processing were investigated.

## 2. Materials and Methods

### 2.1. Antibodies and Chemicals

Rabbit polyclonal antibodies (pAbs) against TrkB (1 : 100), p-TrkB (1 : 100), and Akt (1 : 200) were obtained from Millipore (Billerica, MA, USA). pAbs against p-Akt (1 : 50), Erk1/2 (1 : 100), and p-Erk1/2 (1 : 200) were obtained from Abcam (Cambridge, UK). pAbs PLC*γ*1 and p-PLC*γ*1 (Tyr783) were from Cell Signaling Technology (Danvers, MA). BDNF ELISA kit was from Abcam. K252*α* (inhibitor of tropomyosin receptor kinase B (TrkB)), Hoechst, and bicinchoninic acid (BCA) kit were obtained from Sigma (St. Louis, MO, USA). Alexa Fluor 568-conjugated (1 : 1000) was from Invitrogen (Carlsbad, CA, USA). Peroxidase-conjugated goat anti-mouse (1 : 200) and goat anti-rabbit secondary antibodies (1 : 200) were obtained from Pierce (Rockford, IL, USA).

### 2.2. TBI Model and Treatment

Eight-week male SD rats were from the Chinese Military Academy of Medical Sciences and maintained in the animal center of the Key Laboratory of Neurotrauma Repair in Tianjin, China. All animal experiments were performed according to the “Policies on the Use of Animals and Humans in Neuroscience Research,” which was revised and approved by the Society for Neuroscience in 1995.

Rats were anesthetized with an intraperitoneal injection of pentobarbital sodium solution at a dose of 50 mg/kg body weight and were surgically prepared for lateral fluid percussion brain injury or sham operation as described [16]. Briefly, a 4-mm craniotomy overlying the right parietal cortex (3.6 mm posterior to bregma and 2.5 mm lateral to the midline) was performed. A plastic injury tube was then placed over the exposed dura, bonded by adhesive and dental acrylic, and then allowed to harden. Rats were attached to the fluid percussion injury device (Custom Design and Fabrication, VA, USA) via a female Luer-Lok adapter. Severe brain injury was then induced by the rapid injection of saline solution using a pressure pulse (2.0 atm) into the closed cranial cavity.

Rats were randomly assigned into four groups with different treatment strategies, the sham TBI group (TBI, *n* = 20), TBI group, TBI plus placebo-acupuncture group (*n* = 20), TBI plus acupuncture group (TBI + Acu, *n* = 20), and TBI plus acupuncture plus K252*α* group (TBI + Acu + K252*α*, *n* = 20).

Acupuncture was implemented within 24 h after TBI. Before acupuncture, the rats were immobilized using special cages. The acupuncture needles (40 mm in length, 0.30 mm in diameter, Huatuo Suzhou Medical Instruments Factory, Suzhou, China) were inserted to a depth of 3 mm at the Baihui (DU20), Renzhong (DU26), Hegu (LI4), and Zusanli (ST36). The needles were twisted 2 times/s for 30 s, and the acupuncture treatment lasted 30 min. Animals in the acupuncture group were treated once daily for 14 days. When the acupuncture treatment was implemented, rats in TBI groups without acupuncture were also lightly immobilized in the same way.

For treatment of K252*α*, the rats were anesthetized with injection of pentobarbital sodium solution and placed in a stereotaxic instrument. One hole was made on the opposite side of injury for injection at coordinates −1.0 mm anterior to posterior (AP) bregma and 1.5 mm mid to lateral (ML) and −3.8 mm dorsal to ventral (DV) dura according to the stereotaxic atlas of Paxinos and Watson (Paxinos, 1996). 5 *μ*L of K252*α* (120 nM) was injected into the left side of the lateral ventricle.

### 2.3. Evaluation of Neurological Function

For modified neurological severity score (mNSS), the neurological behavior of rats was assessed at 7 and 21 d after the establishment of the model. The animals were scored for movement, sensation, and reflex. For beam walking test (BWT), the mouse walks directly to the end of the beam (score 0). The mouse walks along the beam with a few slips (score 1). The mouse cannot walk more than a few steps (score 2). The mouse does not move (score 3) [17]. For inclined-grid climbing test, the highest degree of inclination was defined as the ability to maintain body position for 20 s on two separate trials [18]. For Morris water maze test, the rats were trained to find a submerged platform by using a stationary array of cues outside the pool tub. The water was made opaque by using black ink for chiaroscuro. Four spaced trials a day were performed from 14 d to 19 d after TBI. The probe tests were performed with the platform 19 d after TBI and without the platform 21 d after TBI [19].

### 2.4. LTP Measurement

Long-term potentiation (LTP) recording was performed according to the method previously described [20]. Briefly, rats were anesthetized and placed on the stereotaxic instrument. The skull was exposed, and a small hole was made at the contralateral of injury. The stimulating electrode was placed at AP −6.8 mm, ML 4.5 mm, and DV −3.5 mm. The recording electrode was placed at AP −3.5 mm, ML 3.4 mm, and DV −3.5 mm. For each recording experiment, a stable baseline for at least 20 min was required before application of conditioning stimuli. LTP was elicited using high-frequency stimulation consisting of four trains of 50 pulses delivered at 200 Hz with a 2-second interval.

### 2.5. Immunofluorescence

The animals were deeply anesthetized and then transcardially perfused with saline solution, followed by 4% paraformaldehyde solution [21]. The brains were removed and postfixed in paraformaldehyde solution for 12 h. Coronal brain sections were cut with a thickness of 30 *μ*m using a vibrating microtome (Leica VT1000 S, Germany). After blocking endogenous peroxidases and nonspecific binding sites, the sections were incubated with primary antibodies and then Alexa Fluor 568-conjugated secondary antibodies. The images were visualized with a confocal microscope (Leica TCS SP5, Germany).

### 2.6. Western Blotting and ELISA

Western blotting was performed as described previously [22]. Briefly, the protein concentration of tissue homogenates was measured by the BCA kit. Equal amounts of protein were separated by 10% SDS-polyacrylamide gel electrophoresis and transferred to polyvinylidene difluoride membranes. Then the membranes were probed with primary antibodies overnight at 4°C and then incubated with anti-rabbit IgG antibodies conjugated to horseradish peroxidase (1 : 5000) for 1 h at 37°C. Densitometric measurements of band intensities were performed using the Quantity One software (Bio-Rad). The level of BDNF in cortical homogenates was measured by the BDNF ELISA kit according to the manufacturer's instructions.

### 2.7. Statistical Analysis

Data were analyzed using the PSS 15.0 statistical software (SPSS, Chicago, IL, USA). Data were reported as means ± standard deviations (SD). Statistical significance was determined by the one-way ANOVA procedure followed by the LSD post hoc test with 95% confidence.* P* values < 0.05 were considered to be significant.

## 3. Result

### 3.1. BDNF/TrkB Pathway Was Transiently Activated after TBI

**Figure 1:**
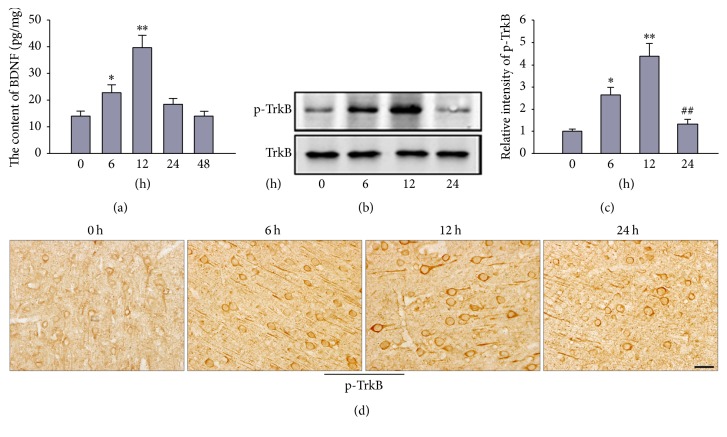
The expression of BDNF and phosphorylated TrkB had been transiently enhanced after TBI. Combined stimulation at the acupoint of Baihui, Renzhong, Hegu, and Zusanli was treated after TBI (once daily for 14 days). The level of BDNF at 0 h, 6 h, 12 h, 24 h, and 48 h after TBI was examined by ELISA kit (a). The phosphorylated TrkB at 0 h, 6 h, 12 h, and 24 h after TBI was detected by western blot (b) and immunohistochemistry (d). (c) is the quantitative analysis of (b). Scale bar, 50 m. ^*∗*^*P* < 0.05 and ^*∗∗*^*P* < 0.01 versus 0 h after TBI. ^##^*P* < 0.01 versus 12 h after TBI.

The modest TBI model was built by fluid percussion instrument. Tissue homogenates and sections were harvested. The level of BDNF was examined by ELISA. The expression of phosphorylated TrkB was detected with western blotting and immunohistochemistry. The results found that the level of BDNF was elevated spontaneously, which reached up to the peak at 12 h and declined to the normal at 48 h after TBI (Figure 1(a)). The phosphorylated level of TrkB reached up to the peak at 12 h and declined to the normal at 24 h after TBI showed by western blot (Figures 1(b) and 1(c)). The phosphorylated TrkB was mainly located in the axon or cell body in pyramidal cells. The result of immunohistochemistry showed the similar trend with the western blot (Figure 1(d)).

### 3.2. The Activation of BDNF/TrkB Pathway after TBI Was Prolongated by Acupuncture

**Figure 2:**
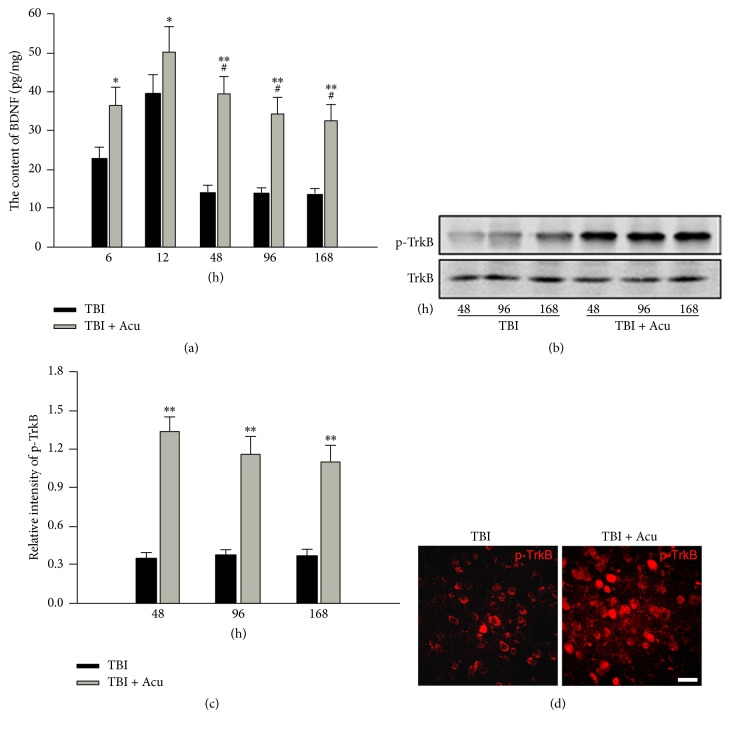
The expression of BDNF and phosphorylated TrkB in acupuncture-treated rats had been persistently enhanced after TBI. The level of BDNF at 6 h, 12 h, 48 h, 96 h, and 168 h after TBI was examined by ELISA kit (a). The phosphorylated TrkB at 48 h, 96 h, and 168 h after TBI was detected by western blot (b) and immunohistochemistry (d). (c) is the quantitative analysis of (b). Scale bar, 50 m. Acu: acupuncture. ^*∗*^*P* < 0.05 and ^*∗∗*^*P* < 0.01 versus TBI group. ^#^*P* < 0.05 versus TBI group at 6 h.

Baihui, Renzhong, Hegu, and Zusanli were selected to acupuncture rats after TBI. The level of BDNF was also examined by ELISA. The expression of phosphorylated TrkB was detected with western blotting and immunohistochemistry. We observed that the level of BDNF had declined to the normal at 48 h in TBI group whereas it still enhanced significantly at 48 h, even at 168 h, in acupuncture group (Figure 2(a)). We also found that the expression of phosphorylated TrkB was significantly increased in acupuncture group compared with TBI group (Figures 2(b)–2(d)). These data showed that acupuncture could prolongate the activation of BDNF/TrkB pathway.

**Figure 3:**
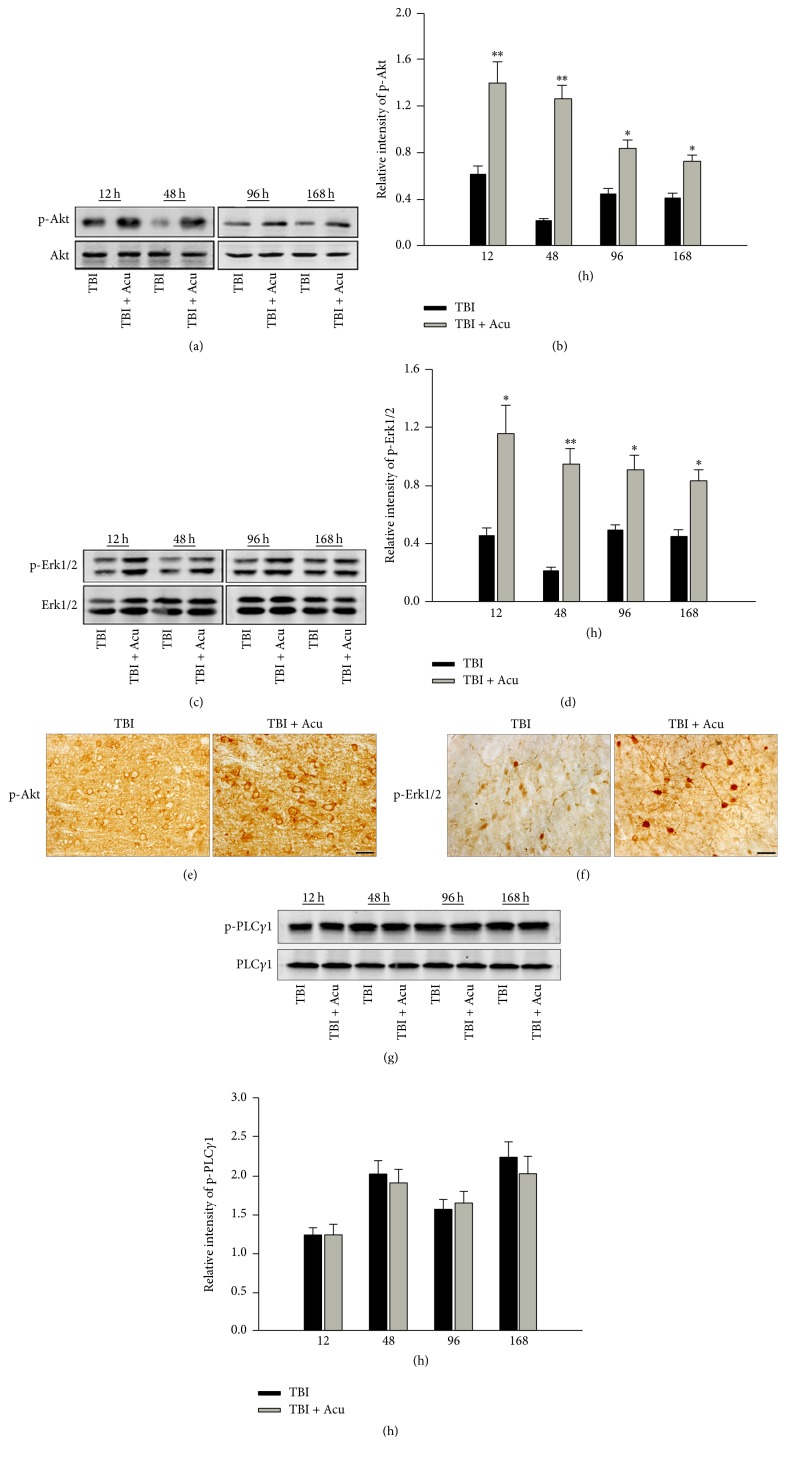
The phosphorylation of downstream molecular BDNF/TrkB signaling pathway was significantly increased in acupuncture-treated rats had been persistently enhanced after TBI. The phosphorylated Akt at 12 h, 48 h, 96 h, and 168 h after TBI was examined by western blot (a) and immunohistochemistry (e). (b) is the quantitative analysis of (a). The phosphorylated Erk1/2 at 12 h, 48 h, 96 h, and 168 h after TBI was examined by western blot (c) and immunohistochemistry (f). (e) is the quantitative analysis of (d). The phosphorylated PLC*γ*1 at 12 h, 48 h, 96 h, and 168 h after TBI was examined by western blot (g). (h) is the quantitative analysis of (g). Scale bar, 50 m. Acu: acupuncture. ^*∗*^*P* < 0.05 and ^*∗∗*^*P* < 0.01 versus TBI group.

Moreover, the downstream molecular of BDNF/TrkB pathway was evaluated by western bolt and immunohistochemistry. Firstly, we examined the activation of Akt and Erk1/2. The results showed that the level of phosphorylated Akt (Figures 3(a), 3(b), and 3(e)) and phosphorylated Erk1/2 (Figures 3(c), 3(d), and 3(f)) was significantly elevated at 12 h, 48 h, 96 h, and 168 h in acupuncture group compared to TBI group, indicating that the extent and time of activated PI3 K-Akt and ras-MAPK signaling pathway were significantly strengthened and elongated in acupuncture group than simple TBI group. Then, we detected the activity of another downstream molecular PLC*γ*1. The result about phosphorylated level of PLC*γ*1 showed no significant difference between TBI plus acupuncture rats and TBI alone rats (Figures 3(g) and 3(h)). The above results suggested that acupuncture could further upregulate the expression of BDNF, activated TrkB by phosphorylation, and trigger the PI3 K-Akt and ras-MAPK signaling pathway.

### 3.3. Blocking BDNF/TrkB Pathway Inhibited Acupuncture-Induced Improvement of Neurological Impairment after TBI

**Figure 4:**
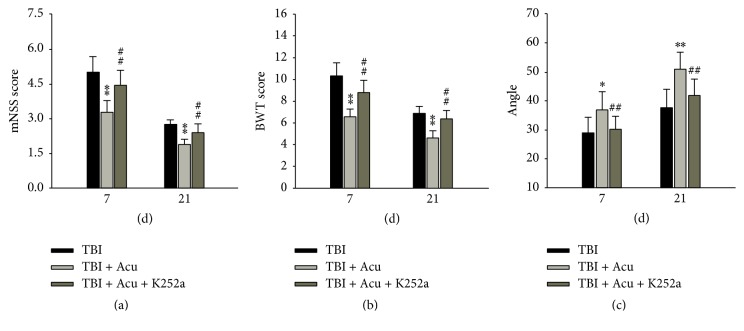
Blocking BDNF/TrkB pathway significantly prevented acupuncture-induced amelioration of neurological deficit. K252*α* (specific inhibitor of TrkB phosphorylation) was injected stereotaxically into lateral ventricle. The mNSS score, BWT score, and climbing angle were assessed at 7 d and 21 d after TBI. Acu: acupuncture. ^*∗*^*P* < 0.05 and ^*∗∗*^*P* < 0.01 versus TBI group. ^##^*P* < 0.01 versus TBI + Acu group.

To further verify the role of BDNF/TrkB signaling in acupuncture repairing TBI, specific tyrosine inhibitor K252a was injected stereotactically into lateral ventricles. The results found that, compared with TBI plus acupuncture group, mNSS, BWT, and angle test were significantly increased (*P* < 0.01) (Figure 4(a)), elevated (*P* < 0.01) (Figure 4(b)), and decreased (*P* < 0.01) (Figure 4(c)) in TBI plus acupuncture plus K252a group.

**Figure 5:**
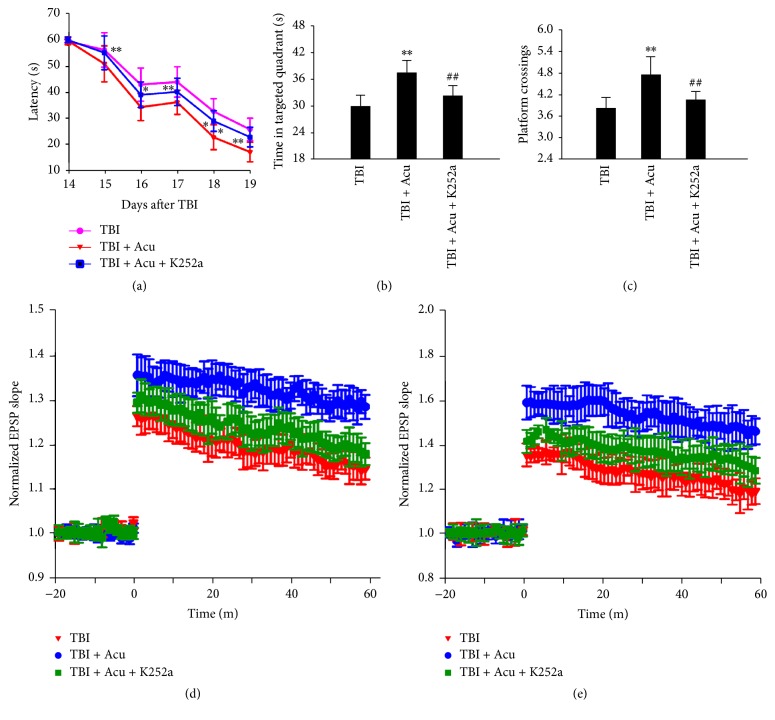
Blocking BDNF/TrkB pathway significantly prevented acupuncture-induced amelioration of cognitive impairment. The rats were trained to remember the hidden platform in the maze from 14 to 19 days after TBI (a). The platform was removed at 21 days, then the time stayed in the target quadrant (b), and the number of crossing platform (c) were examined. (d–f) LTP measurements were performed at 21 days after TBI. Normalized excitatory postsynaptic potential (EPSP) slope (d) and population spike (PS) amplitude (e) were measured. ^*∗*^*P* < 0.01 and ^*∗∗*^*P* < 0.01 versus TBI group; ^##^*P* < 0.01 versus TBI + Acu group.

To evaluate the possible role of acupuncture on the cognitive function, rats after TBI were trained in Morris water maze. We observed that acupuncture dramatically decreased the latency of the rats compared to TBI group (Figure 5(a)). By removing the platform, we also found that acupuncture significantly increased the time in target quadrant and the number of crossing platform compared with TBI group. To assess the role of BDNF/TrkB pathway in amelioration of cognitive deficit induced by acupuncture, we used specific tyrosinase inhibitor K252*α* to block BDNF/TrkB pathway. We found that blocking BDNF/TrkB signaling pathway significantly inhibited the decrease of latency (Figure 5(a)), the increase of crossing number (Figure 5(b)), and the enhancement of time in targeted quadrant (Figure 5(c)) in Morris water maze induced by treating acupuncture.

To investigate the underlying molecular mechanisms of the cognitive amelioration in rats treated with acupuncture, we first explored the synaptic plasticity by examining a LTP paradigm. LTP was analyzed by the measurement of excitatory postsynaptic potential (EPSP) and population spike (PS). We observed that, compared with TBI group, acupuncture inhibited LTP shown by the increased EPSP slope and PS amplitude (Figure 5(d)), demonstrating improvement of synaptic plasticity induced by acupuncture. Moreover, we found that blocking BDNF/TrkB signaling pathway prevented LTP improvement with treatment of acupuncture (Figure 5(e)). These data suggested that the amelioration in memory and synaptic plasticity observed in acupuncture rats mediated via BDNF/TrkB pathway.

## 4. Discussion

With rapid societal development and the accompanying increase in traffic accidents, the rate of TBI has been growing [23, 24]. The presence of neurotrophic factors could facilitate the activation of repair mechanism and stimulation of neurogeneration [25, 26]. Studies found that BDNF can promote the development and survival of neuronal cells and regulate synaptic plasticity and release of neurotransmitters [15]. BDNF exerts substantial protective effects and ameliorates neurological deficits [27, 28]. In this study, rats TBI model was built with fluid percussion injury device. We found that the level of BDNF was elevated spontaneously after TBI and reached up to the peak at 12 h, suggesting that the expression of BDNF enhanced under condition of acute brain injury by self-compensatory regulation, thereby promoting the repair of damaged tissue. However, this enhanced level of BDNF is temporary and quickly declined to the normal at 48 h after TBI. Thus, sustained expression of BDNF is pivotal for the recovery of TBI.

Acupuncture has long been used in China to treat neurological impairment and may be a promising treatment strategy for neurological disorders [29–31]. Here, stimulation at the combined acupoint of Baihui, Renzhong, Hegu, and Zusanli was treated after TBI. We found that BDNF was significantly increased not only immediately after TBI, but also at 168 h after TBI compared with TBI simple group, indicating that acupuncture could persistently enhance the expression of BDNF. The placebo acupuncture group had no obvious change with TBI simple group, which shows the site-specificity of acupuncture-induced effects. BDNF binds to the TrkB receptor and activates of downstream signaling pathways, bringing a series of pathological and physiological changes [32, 33]. Besides BDNF, the level of TrkB was subsequently elevated after acupuncture compared with TBI group, suggesting that BDNF/TrkB may be activated by acupuncture. Then, we examined the downstream molecular of BDNF/TrkB signaling pathways [34]. We observed that both phosphorylated Akt and phosphorylated Erk1/2 were significantly increased showing the activation of BDNF/TrkB pathway.

To further verify that acupuncture improves neurological recovery through activating BDNF/TrkB pathway, specific inhibitor of TrkB K252a to inhibit the phosphorylation of tyrosine was treated by injection stereotaxically into lateral ventricle [35]. We observed that acupuncture could significantly induce the decline of mNSS score, the decrease of BWT score, the increase of climbing angle, the reduce of latency to find platform, the increase of time in targeted quadrant and the increase of numbers crossing platform. We also found that acupuncture could enhance the PS and EPSP of LTP. These data suggested that combined acupuncture improved the neurological function after TBI. However, injection of K252a significantly prevented this acupuncture-induced neurological improvement, indicating that acupuncture promoted the recovery of neurological impairment after TBI by activating BDNF/TrkB signaling pathway.

Acupuncture has been demonstrated to be effective worldwide in treating many disorders and endorsement. However, the lack of general mechanism brings considerable controversy for the acupuncture in evidence-based medicine. In this study, we verified that acupuncture continually promoted the production of BDNF, the enhanced expression of TrkB, activation the downstream signaling pathway, and thereby the recovery of neurological deficit, which provided more molecular mechanism for understanding traditional therapy of acupuncture.
